# Propagation of extended fractures by local nucleation and rapid transverse expansion of crack-front distortion

**DOI:** 10.1038/s41567-023-02365-0

**Published:** 2024-01-29

**Authors:** T. Cochard, I. Svetlizky, G. Albertini, R. C. Viesca, S. M. Rubinstein, F. Spaepen, C. Yuan, M. Denolle, Y-Q. Song, L. Xiao, D. A. Weitz

**Affiliations:** 1 National Key Laboratory of Petroleum Resources and Engineering, China University of Petroleum, Beijing, China; 2 School of Engineering and Applied Sciences (SEAS), Harvard University, Cambridge, MA USA; 3 Faculty of Engineering, University of Nottingham, Nottingham, UK; 4 Department of Civil and Environmental Engineering, Tufts University, Medford, MA USA; 5 The Racah Institute of Physics, The Hebrew University of Jerusalem, Jerusalem, Israel; 6 Department of Earth and Planetary Sciences, Harvard University, Cambridge, MA USA; 7 Earth and Space Sciences, University of Washington, Seattle, WA USA; 8 Athinoula A. Martinos Center for Biomedical Imaging, Department of Radiology, Massachusetts General Hospital, Charlestown, MA USA; 9 Department of Physics, Harvard University, Cambridge, MA USA; 10 Wyss Institute for Biologically Inspired Engineering, Harvard University, Boston, MA USA

**Keywords:** Applied physics, Materials science

## Abstract

Fractures are ubiquitous and can lead to the catastrophic material failure of materials. Although fracturing in a two-dimensional plane is well understood, all fractures are extended in and propagate through three-dimensional space. Moreover, their behaviour is complex. Here we show that the forward propagation of a fracture front occurs through an initial rupture, nucleated at some localized position, followed by a very rapid transverse expansion at velocities as high as the Rayleigh-wave speed. We study fracturing in a circular geometry that achieves an uninterrupted extended fracture front and use a fluid to control the loading conditions that determine the amplitude of the forward jump. We find that this amplitude correlates with the transverse velocity. Dynamic rupture simulations capture the observations for only a high transverse velocity. These results highlight the importance of transverse dynamics in the forward propagation of an extended fracture.

## Abstract (web summary)

Understanding the three-dimensional nature of fracture formation and dynamics is challenging. Experiments now show that a fracture front, after originating at a particular locus in a material, propagates jump-wise and expands transversely at high speed.

## Main

Fractures occur at all length scales, from those that are familiar to us, such as the breaking of a wine glass or a cell-phone screen, to those that are geological, such as earthquakes^1^ or the calving of glaciers^2^. Even with this wide range, some phenomena transcend all length scales and provide a basis for understanding the fundamental features of fractures. In the simplest case, in two dimensions, the fracture surface is approximated by a line and the fracture front is a point. By contrast, an extended fracture in three dimensions is a surface that terminates on a line, the crack front. When this line is straight and the fracture surface is a plane, a two-dimensional (2D) projection adequately describes the full three-dimensional (3D) dynamics^3^. This 2D approximation of fracturing is well understood: the stress at the crack tip is described by a universal singularity, and the balance between the energy dissipated during fracture formation and the stored elastic energy determines an equation of motion for the fracture that provides a complete description of its onset and propagation^4^. However, a more realistic fracture of a material in 3D presents a plethora of complex behaviours, which have been widely studied yet remain poorly understood at a fundamental level^3^. Any distortion of the crack front out of the fracture plane alters the singularity of the stresses and can lead to complex structures imprinted on the fracture surface, as inferred by port-mortem observations^5–10^. Even any in-plane distortion of the crack front from a straight line leads to complex propagation dynamics due to the interplay between the singularity and the curvature of the line. Such distortions can occur during slow, quasi-static propagation^11^, for example, when a steadily propagating front encounters heterogeneities in the material fracture resistance, which impede the local advance of the crack^12–15^. Distortions may also occur during more rapid propagation, during which material inertia leads to wave-mediated interactions along the fracture front^14,15^, so that local asperities lead to the remarkably rapid transverse propagation of these crack-front distortions^16–23^. More generally, material heterogeneities can result in the irregular propagation of a crack front in both space and time. Even in the absence of local heterogeneities, simulations and theory suggest that it is energetically favourable for crack propagation to occur in crystalline materials through a localized bond rupture followed by sequential lateral motion of atomic kinks along the crack front rather than through a uniform advance^24–30^. However, detailed experimental observations of the dynamics of crack propagation in 3D and the effects of any distortions are difficult and have yet to be reported. Any distortion of a crack front precludes the use of the approximation of the 2D projection^11^; thus, to fully understand the onset and propagation of fractures, the role of the dynamics of any distortions must be investigated.

In this Article, we examine the onset, propagation and arrest of an extended fracture. We visualize the dynamics and show experimentally that distortions of the crack front are essential. The forward propagation of a fracture occurs through an initial rupture, nucleated at some localized position, which induces high local curvature of the crack front. This is followed by the rapid transverse expansion of the crack front. The transverse velocity depends on the amplitude of the initial distortion, which is controlled by the loading at the crack front. For a large distortion, it can be as fast as the velocity of the Rayleigh wave, whereas for smaller distortions, the transverse velocity is reduced. Interestingly, dynamic rupture simulations reproduce the experimental observations only when the transverse velocity is that of the Rayleigh wave. These results highlight the importance of distortions and their dynamics in fracture propagation.

**Fig. 1:**
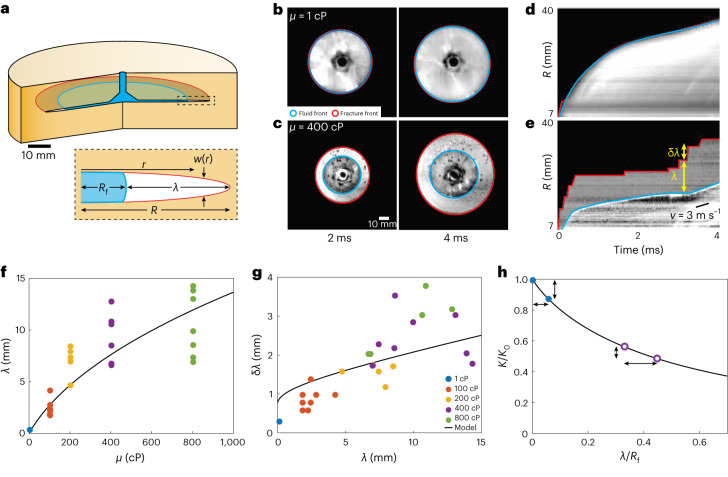
Experimental system and properties of extended fractures. **a**, Top, experimental set-up showing a penny-shaped growing fracture with the fluid (blue) lagging the fracture front (red). Bottom, cross section of the fracture and injected fluid. **b**,**c**, Images of the fracture 2 ms (**b**) and 4 ms (**c**) after crack initiation. The fracture front (red lines) leads the fluid front (blue lines) for both *μ* = 1 and 400 cP. **d**,**e**, Kymographs from the edge of the injector in the north-east direction showing the amplitudes of the fluid front (blue lines) and fracture front (red lines). The amplitude of the fracture follows stick–break motion and exhibits abrupt jumps followed by long times with no motion, for both *μ* = 1 Cp (**d**) and 400 cP (**e**). **f**, Measured dependence of *λ* on *µ* compared to the model predictions. **g**, Measured values of δ*λ* as a function of *λ* compared to the predictions of a model based on linear elastic fracture mechanics. The value of the viscosity for each data point is denoted by its colour and is given in the legend. **h**, Variation of stress intensity factor with lag, normalized by the radius of the fracture. The fracture is initiated at a value of *K* slightly larger than the value when it arrests, allowing it to propagate a small distance much faster than the fluid, thereby increasing the lag by a small amount, as shown by the two circles with the arrows. When the lag is smaller, the same δ*K*/*K*_0_ leads to a smaller increase in the lag, as shown by the two solid points and the arrows on the left.

We use an experimental system developed specifically to probe the initiation and propagation of an extended fracture. To attain an uninterrupted, extended fracture, we study a penny-shaped crack in a cylindrical geometry. To precisely control the loading conditions, we induce the fracture through fluid injection and vary the fluid viscosity^31–36^. To probe the propagation of the full fracture front, we use a transparent material and image the crack motion with a high-speed camera. We use a 10-cm-diameter cylinder made of stereo-lithographically 3D-printed optically clear polymethylmethacrylate (PMMA). Fluid is injected through a hole in the centre of the sample, and a small notch initiates the crack (Fig. 1a). To control the loading, we vary the viscosity *μ* of the fluid that drives the fracture; to accomplish this, we use water-glycerol mixtures with different ratios. The fluid is injected with a high-pressure syringe pump operated at a constant flow rate of 0.3 ml min^−1^. The pressure is measured with a gauge on the syringe pump that has a response time of 100 ms. The pressure increases linearly with time for 250 s, whereupon the crack is initiated at a pressure of roughly 40 MPa, leading to a sudden drop in pressure. The subsequent complete fracturing of the sample is so rapid that no additional fluid is injected by the pump. At these pressures, the fluid is compressed, and the propagation is driven exclusively by the expansion of the fluid and any small compliance within the experimental system. We record 500 × 500 pixel images at 100,000 frames per second using a high-speed camera. The fluid is dyed with fluorescein, and the sample is illuminated with an intense blue light-emitting diode ring, which enhances the contrast between the fluid and the solid to improve the visibility of the fracture.

To explore fracture behaviour under different loading conditions, we vary the fluid viscosity. We enhance the visibility of the expanding fracture by subtracting the image just before the fracture is initiated from all later images. Thus, the fracture appears as a bright, circularly expanding ring in Fig. 1b,c, whereas the fluid appears as a darker inner circle in each image. We identify both the fluid front (blue) and fracture front (red) as they propagate radially outward. When the fluid is pure water with a viscosity of 1 cP, the fluid front coincides with the fracture front and they both expand outwards simultaneously, as shown in the series of images in Fig. 1b. To change the loading conditions, we increase the fluid viscosity. This induces a distinct lag between the two fronts, which allows us to vary the distance between the position where the stress is applied and the fracture front, as shown for *μ* = 400 cP in Fig. 1c. To characterize the propagation, we construct kymographs by plotting the intensity of each image along a single radial direction as a function of time. For the low-viscosity fluid, the fluid front (blue) is indistinguishable from the fracture front (red), as shown in Fig. 1d. By contrast, for the loading with the high-viscosity fluid, there is distinct lag between the two fronts, as shown in Fig. 1e. Interestingly, the propagation of the fracture front is discontinuous with long pauses of no motion followed by rapid forward motion, whereas the propagation of the fluid front is continuous. We call this a stick–break instability. Although reminiscent of stick–slip-like behaviour for mode I fracture under remote loading conditions^5,37^, such discontinuous motion has not been observed before for fluid-driven fracture. The time interval between the break events varies and can be as long as a millisecond. The forward velocity during the break events is very high, faster than our spatio-temporal resolution. The length of the break δ*λ* varies and can be as large as 5 mm, in which case the radial velocity is at least 500 m s^−1^. The lag between the fluid and the fracture fronts *λ* varies with time as breaks occur but is persistently between 5 and 7 mm (Fig. 1e). The time-averaged radial velocities of each front are similar and are approximately 3 m s^−1^. Thus, the break portion of the stick–break behaviour leads to very rapid motion of the fracture, and it outruns the slower motion of the fluid whose pressure drives the fracture. The motion of the fracture then pauses during the stick portion, and suddenly resumes at the next break. This behaviour is reminiscent of a nucleation process that instigates a break. Although the instantaneous fracture velocity can be quite high during the break portion of the motion, the average fracture velocity is much lower because of the long pauses during the stick portion. Earlier studies of fracture propagation did not observe the stick–break instability and, thus, underestimated the true velocity of the fracture front^10,35^.

The stick–break instability is a consequence of the loading condition for the fracture. Similar behaviour should occur whenever there is a gap between the applied stress and the fracture tip. In our experiments, the fluid lag can be controlled by the fluid viscosity, with *λ* increasing with *μ*, as shown by the data points in Fig. 1f. In turn, there is a strong correlation between the values of δ*λ* and *λ*, with the length of the break increasing with that of the lag, as shown in Fig. 1g. To determine the origin of this behaviour, we consider the stress intensity factor *K* of a fluid-driven penny-shaped fracture (Methods). Here, *λ* is the difference between the crack radius *R* and the position of the front of the pressurized fluid *R*_f_, as shown in Fig. 1a (bottom). The stress intensity factor decreases with increasing lag, as seen in Fig. 1h, which is a plot of *K*/*K*_0_ as a function of *λ*/*R*_f_, where *K*_0_ is the value of *K* at *λ* = 0. We assume that the fluid front is stationary during the very short time of a crack jump. Thus, *K* decreases as the crack propagates, and the crack stops when *K* reaches a characteristic value *K*_c_, which is the material toughness^38^. To account for the nucleation behaviour apparent in the stick–break instability of the fracture motion, we assume that the crack is initiated at a somewhat higher value, *K*_c_ + δ*K*_c_ (refs. ^5,39^), which corresponds to a smaller lag, *λ* − δ*λ*. Because d*K*(*λ*)/d*λ* increases with decreasing *λ*, the length of the break must decrease as the lag decreases, provided δ*K*_c_/*K*_c_ remains constant, as can be seen by comparing the open circles and the solid points in Fig. 1h. We calculate the dependence of δ*λ* on *λ* assuming δ*K*_c_/*K*_c_ *≈* 12% (Methods) and obtain good agreement with the data, as shown by the solid line in Fig. 1g. By incorporating the fluid flow in our description of the fracture (Methods), we determine the *μ* dependence of *λ* and obtain good agreement with the measured behaviour, as shown in Fig. 1f.

**Fig. 2:**
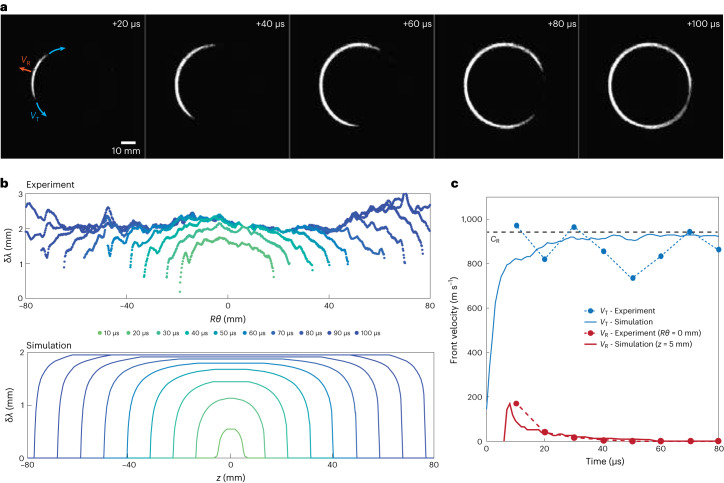
Details of propagation of extended fracture for a single nucleation event. **a**, Differential images of the time evolution of a single nucleation event obtained by subtracting the image just before the nucleation. Each image is 20 μs later than the previous one. The series shows the rapid transverse propagation of the fracture following the initial radial expansion shown in the first image. The radial (*V*_R_) and transverse (*V*_T_) velocities are shown by the arrows. **b**, Time evolution of the fracture front plotted as a function of radial distance from the point of initial nucleation. The graph shows the localized initial fracture in the radial direction followed by rapid expansion transversely for the experiments (top) and the simulations (bottom). **c**, Radial velocity of the fracture front at the point of nucleation (*Rθ* = 0 mm and *x* = 5 mm). There is a rapid initial jump followed by a much slower decay, for both the experiment and simulations (red line). The transverse velocity of the fracture front, measured at δ*λ* = 1.5 mm, is high as the fracture propagates transversely along the full perimeter, for both experiments and simulations (blue lines). The transverse velocity is remarkably close to the Rayleigh-wave velocity *C*_r_ shown by the dashed line.

Although the model provides excellent agreement with the data, it intrinsically assumes a circularly symmetric interface and, therefore, inherently ignores any possible distortion of the fracture front. As the fracture propagates through the stick–break instability, we can investigate many instances of the propagation of an extended fracture front from a stationary crack and can thereby determine exactly how fracture advance begins. We focus on the behaviour when the applied load is far from the fracture front, and thus, the lag is large. We, therefore, consider the data for *μ* = 400 cP. Careful inspection reveals that each advance of the fracture front is not uniform along its full extent. Instead, the initial nucleation of any break event is extremely rapid yet seems to be spatially localized, and thus, the fracture front must be locally distorted, as seen in Supplementary Video 1. To directly observe the advance, we examine the differential growth of the single stick–break instability highlighted by yellow arrows in Fig. 1e. We subtract the image just before the initial rupture from each of the subsequent images. The forward advance of the fracture front does, indeed, begin at some localized region. It then rapidly spreads transversely along the full extent of the front, as shown by the sequence of images separated by 20 μs in Fig. 2a. The fracture propagates transversely fully around half the perimeter, corresponding to 9 cm, in roughly 100 μs. This represents an exceptionally fast velocity of 900 m s^−1^, comparable to the Rayleigh-wave speed, *C*_R_ = 940 m s^−1^, as determined by acoustic methods (Supplementary Fig. 1).

To follow the time evolution of the profile of the crack front, we detect the edges of the bright region and measure δ*λ* in each image as a function of the transverse distance along the circumference *Rθ*, where *R* is the radius of the fracture measured from the injection point and *θ* is the angle measured from the nucleation position. The first image of the fracture front exhibits a very strong distortion where the initial rupture occurs. The front is sharply curved with the edges nearly perpendicular to the transverse direction, as shown by the green curve in Fig. 2b (top). The shape of the distortion of the fracture front remains the same as it spreads transversely. The propagation of the flat region in the forward (radial) direction slows and stops after three frames (30 µs) whereas the perpendicular edges expand at a nearly uniform rate, as shown in Fig. 2b (top). To quantify the motion, we determine the velocity of the fracture front. Because of the limited time resolution, we can determine only a lower bound for the velocity of the forward motion. However, at any point, there is a rapid initial forward velocity followed by much slower motion, as shown, for example, at the nucleation location, *Rθ* = 0 mm (red), in Fig. 2c. The transverse velocity can be measured more precisely. We plot the time the fracture front reaches 1.5 mm, about 75% of the total jump amplitude, as a function of radial distance (solid blue points in Fig. 2c). The transverse velocity is comparable to that of the Rayleigh wave indicated by the black dashed line in Fig. 2c. The behaviour is fundamentally the same for each break event. The initial fracture advance is through nucleation at some localized position, followed by an ultra-fast transverse expansion around the circumference to fully extend the fracture.

**Fig. 3:**
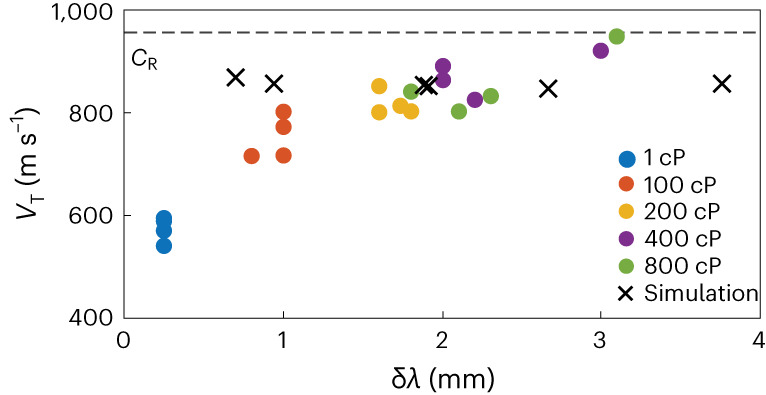
Transverse velocities. Measured transverse velocity as a function of δ*λ* for different viscosities of the driving fluid (circles). For comparison, simulated velocities are also shown (crosses). The velocity of the Rayleigh wave is shown by the dashed line.

Similar behaviour is observed for all loading conditions. For all values of the lag, an analysis using differences between subsequent images clearly shows that fractures propagate through a stick–break motion, nucleated at a single point, and followed by rapid transverse propagation (Supplementary Video 3). The transverse velocity is very fast, comparable to the Rayleigh-wave speed of the material provided that δ*λ* > 2 mm. Interestingly, however, there is a very pronounced decrease in transverse velocity *V*_T_ when δ*λ* approaches zero, as shown in Fig. 3.

To gain insight into the behaviour of the transverse propagation, we simulate the development of a spatially extended planar fracture in a 3D linear elastic medium using a spectral boundary integral method (Methods)^40^. The extended fracture front is initially straight instead of circular. We use values for the material density, elastic moduli and *K*_c_ that mimic those in the experimental system. We consider the propagation of a brittle crack. The crack-tip singularity is regularized by introducing a cohesive zone of size *X*_C_, which is much smaller than all other length scales. The simulation follows the same strategy as the one used to account for the experimental data in Fig. 1. We ignore the fluid itself and assume that the applied pressure is constant beyond a fixed distance from the crack tip, thereby introducing the lag. The initial condition consists of a crack with applied pressure such that it is in equilibrium at half-length *R* with toughness *K*_c_ + δ*K*_c_. However, the toughness ahead of the crack front is reduced to *K*_c_, so that the crack is initially in an unstable equilibrium. We nucleate the instability by reducing the toughness at a localized region along the crack front to *K*_c_.

In our simulation, we use *λ* = 8 mm, which yields a jump size of δ*λ* = 2 mm, consistent with the experiment as shown in Fig. 1g. Once initiated, the fracture propagates dynamically in the transverse direction (Supplementary Video 4 and Supplementary Fig. 5), and the profile of the simulated fracture front matches that of the experiment, as shown by the fracture-front profiles in Fig. 2b (bottom). The velocity in the forward direction reproduces the experimental observations, with an initial rapid rise followed by a deceleration, as shown by the solid lines for *z* = 5 mm (red) in Fig. 2c. Moreover, the simulated transverse velocity is asymptotically very close to the Rayleigh-wave speed, as shown by the blue solid line in Fig. 2c. Consistent with the experiment, in the simulation the jump size increases with the lag. For all values δ*λ* ≥ 2 mm, the transverse velocity is nearly that of the Rayleigh-wave speed in agreement with experiment. By contrast, for smaller δ*λ*, the simulation does not capture the decrease in the transverse velocity observed in the experiment, as shown in Fig. 3. Because the simulation is based on the brittle fracture framework, this discrepancy suggests that there is a deficiency in this framework for short jump sizes. In the simulation, the deficiency with the brittle fracture framework occurs when the jump size becomes comparable to the cohesive zone. Although we have not directly measured the length of the cohesive zone in our samples, it is not unreasonable to expect it to be comparable to the very short δ*λ* measured for water, which is less than 200 µm and is limited by the resolution of our measurement. Alternatively, the discrepancy may arise because the flow of the water over the short length of the jump is sufficiently fast that fluid flow should be explicitly included.

**Fig. 4:**
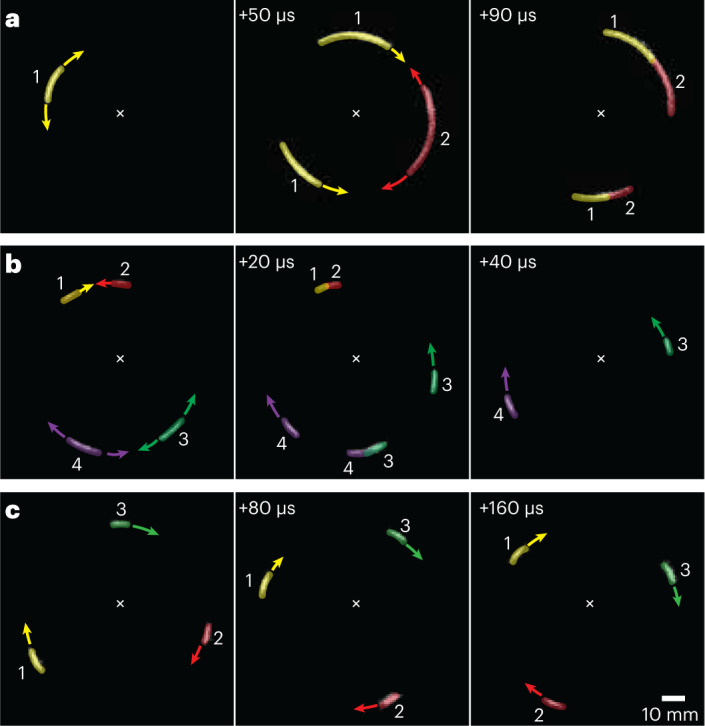
Differential images of local nucleation and transverse propagation of break events. As the viscosity decreases the time between nucleation events becomes shorter than the time for the fracture to traverse half the circumference and, therefore, several nucleation events are observed nearly simultaneously. **a**, Two counter-propagating transverse fractures from earlier nucleation events (1) at t = 0 μs and (2) at t = 50 μs merge and stops at t = 90 μs. **b**, Four counter-propagating transverse fractures from earlier nucleation events (1 and 2) and (3 and 4) at t = 0 μs merge and stops respectively at t = 20 μs and t = 40 μs. **c**, Several fracture events occur nearly simultaneously, the transverse propagation from each break is only in a single direction leading to a continuous, simultaneous propagation of several transverse fractures in the same direction.

By examining differential images in the videos, we determine the effect of the loading conditions on the length scale of the jumps. For large lags, the nucleation events are well separated, and the fracture propagates around the full circumference well before the next nucleation event occurs. By contrast, for smaller lags, the time between the nucleation events decreases markedly and the fracture cannot propagate fully around the circumference before subsequent nucleation events occur. A typical example is shown for *μ* = 100 cP. One nucleation event occurs and propagates about a third of the circumference, whereupon a second nucleation event occurs. Thereafter, the two counter-propagating transverse fractures merge and stop when they meet, as shown in Fig. 4a. This behaviour becomes even more pronounced as *λ* decreases further. For *μ* = 1 cP, additional nucleation events occur well before the transverse propagation of the fracture extends to the full circumference. For example, an instance when two counter-propagating transverse fractures from earlier nucleation events (1 and 2) merge and stop occurs at the same time as two new nucleation events (3 and 4) occur as shown in Fig. 4b. For these new events, the two closest counter-propagating transverse fracture fronts meet and merge, leaving only two counter-propagating transverse fracture fronts remaining, as shown in Fig. 4b and in Supplementary Video 3.

To account for these observations of the *λ* dependence of the intervals between nucleation events, we compare the average stick time ${\tau }_\mathrm{N}=\delta \lambda/v$ to the propagation time required for a transverse fracture to travel around half the circumference ${\tau }_\mathrm{T}=\pi R/{V}_\mathrm{T}$. The average forward velocity of the fracture *v* is determined by the loading rate and is set by the average velocity of the fluid expansion, which is roughly independent of viscosity. Therefore, *τ*_N_ decreases with *λ* as δ*λ* decreases. By contrast, *τ*_T_ increases with viscosity since *V*_T_ decreases with *λ*. This variation accounts for the observed behaviour of several transverse fractures propagating simultaneously (Supplementary Video 2). In fact, when *τ*_T_ is much larger than *τ*_N_, several fracture events occur nearly simultaneously and we observe cases in which the transverse propagation from each break is only in a single direction. Thus, there is a continuous, simultaneous propagation of several transverse fractures in the same direction, as shown in Fig. 4c.

The circular geometry studied in this Article is key for the investigation of extended fractures because of the lack of edges. Thus, no matter where the initial nucleation occurs, it is in the middle of the fracture front. Moreover, the control afforded by fluid-driven fracture enables us to precisely vary the loading conditions and, hence, the distance between the applied stress and the fracture front. Furthermore, a fluid-induced fracture is intrinsically unstable and propagates through a stick–break instability. We exploit these features to demonstrate that crack motion starts with a localized nucleation event followed by a very rapid transverse expansion. Because the fracture is always extended, this behaviour should be ubiquitous and describe the propagation of any fracture. Our results provide a basic understanding of the fundamental nature of fracture propagation in realistic, 3D geometries. Observations of extended fractures for which evidence of irregular crack propagation was inferred from lower-resolution imaging and postmortem studies of the fracture surface^5^ likely point to a similar mechanism. Our results should also be applicable to crystalline materials, which could be used experimentally to validate our simulations and theoretical predictions that suggest that the process of local nucleation and transverse propagation is an energetically efficient route for crack advance in lattice models^14–30^. The same behaviour should occur for fractures at all length scales, from the cracking of windshields and cell-phone screens to in-ground resource recovery, CO_2_ sequestration and geothermal energy. These observations may also provide insights into the behaviour of other classes of fracture propagation, such as earthquakes, as, for example, microearthquakes suggest that localized propagation is distributed over large portions of the fracture front^41^.

## Methods

### Sample preparation and static mechanical properties

Each sample was 3D-printed stereo-lithographically from an optically clear PMMA based resin (clear resin, Formlabs). A sample was cylindrical with a diameter of 10 cm and height of 3.2 cm. A hollow fluid injector was in the centre of the 3D-printed design. The injector consisted of a cylinder of 3.8 mm in diameter and height of 16 mm and was connected to a conic-shaped fracture initiation site of diameter 15 mm at its bottom and of height 3 mm (Fig. 1a). The density of a printed sample was *ρ* = 1,200 kg m^*−*^^3^. The Young’s modulus of the material, *E* = 1.6 GPa, was measured using ASTM 399 testing. The fracture toughness, ${K}_\mathrm{c}=0.75\,\mathrm{MPa}\,\sqrt{\mathrm{m}}$, was measured with ASTM 638 testing. Both tests used a sample of thickness 24 mm and various loading rates ranging from 0.1 to 10 mm min^−1^.

### Measurements of dynamic elastic moduli

We carried out a drop ball test on a 3D-printed cube of dimensions 140 × 140 × 50 mm. Four acoustic emission sensors (KRNBB-PC) were placed in line along the centre of the surface of the sample and separated from each other by 15 mm. A 1-mm-diameter metal ball was thrown from a height of 20 cm, 15 mm away from the first sensor, as shown in Supplementary Fig. 1a,b. Due to this impact, an acoustic wave consisting of a pressure wave and a surface acoustic wave (or Rayleigh wave) was emitted and recorded successively by the four sensors. By looking closely at the signals, we could differentiate the two components of the acoustic wave, as shown in Supplementary Fig. 1c. From the consistent delay of 6.5 µs for the signal measured by each sensor, we estimated that the p-wave velocity was close to *C*_P_ = 2,300 m s^−1^, as shown in Supplementary Fig. 1d (top). The Rayleigh wave resulting from the impact was observed to have a consistent delay of 16 µs, as shown in Supplementary Fig. 1d (bottom). Thus, we estimated the Rayleigh-wave velocity *C*_R_ = 940 m s^−1^. The inferred shear-wave velocity was *C*_S_ = 1,000 m s^−1^. The dimensions of the sample were chosen carefully to avoid any superposition of the first reverberated p wave on one of the faces of the sample with the surface acoustic wave, which was observed at later time. From the measurements of the wave speeds, we obtained the high-frequency Poisson ratio *ν* = 0.38 and Young’s modulus *E* = 3.3 GPa.

### Relative contributions to injection compliance

We measured the total compliance (*U*) of the injection system, which relates the increments of injected fluid volume (*V*_f_) to those of pressure (*p*_f_) before the fracture initiated:

1 $$\frac{\mathrm{d}{V}_\mathrm{f}}{\mathrm{d}t}=U\frac{\mathrm{d}{p}_\mathrm{f}}{\mathrm{d}t}.$$

The total compliance is the sum of the compliance of the apparatus (*U*_a_) plus the compliance of the fluid it contains (*U*_f_): *U* = *U*_a_ + *U*_f_. We measured $U=2.3\times{10}^{-2}$ ml MPa^−1^, and we calculated the compliance of the fluid from *U*_f_ = *βV*_f_, where $\beta =4.6\times{10}^{-10}\,{\mathrm{Pa}}^{-1}$ is the isothermal compressibility of water at 25 °C and where *V*_f_ = 35 ml, including 20 ml in the pump cylinder and 15 ml in the tubing and connections. Therefore, we estimated that *U*_f_ is 70% of *U*, so that water compressibility was the major source of injection compliance. We consider the compressibility only of the water because the fluid entering the fracture (pure water or water-glycerol mixtures) was added only in the injector within the 3D-printed sample, which is only about 2 ml and negligible compared to *V*_f_. Thus, we could neglect the slight difference in the compressibilities of the water and the glycerol mixtures.

The compliance *U* provides a characteristic length scale for a fracture driven by fluid expansion (*UE*′)^1/3^, where *E*′ is the normalized Young’s modulus ${E}^{{\prime} }={E}/(1-{\nu }^{2})$ and *ν* is Poisson’s ratio^35,42^. This length was 4.7 cm and was comparable to the sample radius of 5 cm, suggesting that the source of fluid volume driving the fracture resulted from system compliance and not from the constant rate of injection. This is consistent with the observation of a negligible volume introduced by the pump during the very short time of the complete fracture propagation.

### Modelling the jump amplitude

To gain insight into the stick–break instability, we modelled the behaviour of the fracture using linear elastic fracture mechanics. To make the model more tractable, we ignored the angular dependence and considered a circularly symmetric, penny-shaped fracture such that the difference between the position of the fracture front *R*, as measured from the injection point, and the fluid front *R*_f_ was the lag *λ*, as shown in Fig. 1a (bottom). We neglected the very small pressure of the vapour in the lag region and approximated the fluid pressure with a linear profile $P(r)={P}_{0}(1-r/{R}_\mathrm{f})$, where *P*_0_ is the applied pressure at the inlet. The stress intensity factor *K* is given by^31^

2 $$K=\frac{2}{\sqrt{\pi R}}{\int }_{0}^{{R}_\mathrm{f}}\frac{P(r)}{\sqrt{{R}^{2}-{r}^{2}}}{r\,\mathrm{d}r},$$

where the integration is limited to the fluid region. The stress intensity factor decreased with increasing lag, as seen in Fig. 1h, which is a plot of *K*/*K*_0_ as a function of *λ*/*R*_f_, where *K*_0_ is the value of *K* at *λ* = 0.

We assumed that the fluid front is stationary and that the pressure profile did not change during the very short time of crack propagation. To account for the stick–break nature of the propagation, we assumed that the nucleation of the crack occurred at a somewhat higher value of *K* than did the arrest. Therefore, we took *K*_c_ to be defined by the value of *λ* at which the crack arrested and assumed that crack nucleation occurred at a slightly larger value *K*_c_ + δ*K*_c_, which corresponds to a smaller value *λ* − δ*λ*. This assumes that the crack stopped when the energy released per unit propagation of a crack became smaller than the fracture energy. The jump amplitude is calculated by

$$\delta \lambda =-\left(\frac{\mathrm{d}K(\lambda )}{\mathrm{d}\lambda }\right)^{-1}K(\lambda )\frac{\delta {K}_\mathrm{c}}{{K}_\mathrm{c}},$$

with $\delta {K}_\mathrm{c}/{K}_\mathrm{c}\approx0.12$. The assumption of a linear gradient of pressure may be modified at low viscosities^43^. However, the slight modification of the predicted *λ* dependence of δ*λ* is still in good accord with the experimental data, as shown in Supplementary Fig. 2.

### Modelling the average lag

The crack opening in the vicinity of the tip *w*(*r*) is dominated by a universal square-root form:

3 $$w(r)=\sqrt{\frac{32}{\pi }}\frac{K}{{E}^{{\prime} }}\sqrt{R-r}.$$

To account for the viscosity dependence of *λ*, we incorporated fluid flow in our description of the fracture. We described the fluid flow with the Poiseuille equation:

4 $$q=-\frac{{w}^{3}}{12\mu }\frac{\partial P}{\partial r}.$$

As *q* = *wv* at the fluid front, which moved at a velocity, *v*, we obtained *w* from equation (3) and ∂*P*/∂*r* from equation (4), enabling us to determine the *μ* dependence of the lag:

5 $$\frac{\lambda }{R}=1-\frac{{R}_\mathrm{f}}{R}=\frac{12\sqrt{\pi }}{16}\frac{\mu v{E}^{{\prime} 2}}{{K}_\mathrm{c}^{3}/\sqrt{R}}\frac{{R}_\mathrm{f}}{R}{\int }_{0}^{{R}_\mathrm{f}/R}\frac{(1-x)}{\sqrt{1-{x}^{2}}}{x\,\mathrm{d}x}.$$

Interestingly, we found that the average velocity of the fluid front depended only weakly on *μ*. Therefore, we used *v* = 3 m s^−1^. Because the fracture propagated at high velocities, we accounted for the frequency dependence of the elastic modulus and used the acoustic measurements to determine *E*′ = 3.8 GPa. We were unable to experimentally determine the corresponding value of *K*_c_ for these frequencies. We, therefore, used *K*_c_ = 1 MPa m^1/2^. Both the value of *E*' measured from the acoustics and the value of *K*_c_ used in solving equation (5) are larger than the values measured experimentally with a tensile test, which was a quasi-static measurement (Supplementary Fig. 3). This frequency-dependent increase in each value was expected^44^.

### Spectral boundary integral simulations

The elastic moduli and critical stress intensity factor corresponded to those in the experimental system. We neglected viscous dissipation and considered the propagation of a planar crack within an unbounded linear elastic medium (Fig. 1a). We solved the 3D electrodynamic equations using a spectral boundary integral method^40^ implemented in the open-source software Uguca^45^. This spectral formulation implies that crack propagation occurs along the *x*–*z* plane. There were periodic boundary conditions along *x* and *z*, whereas the domain was unbounded along *y*. Hence, we considered short times, before any interaction of the crack. Waves were radiated with the *x* boundaries.

The crack was modelled using a cohesive law^46,47^ that relates the material tensile strength *τ*_s_ to the crack opening displacement ${\underline{u}}_{y}$. For simplicity, we assumed a linear law:

6 $${\tau }_\mathrm{s}\left({\underline{u}}_{y}\right)=\begin{cases}{\sigma }_\mathrm{Y}\left(1-\frac{{\underline{u}}_{y}}{{\delta }_\mathrm{c}}\right),& \mathrm{for}\;\frac{{\underline{u}}_{y}}{{\delta}_\mathrm{c}} < 1,\\0,&\mathrm{for}\;\frac{{\underline{u}}_{y}}{{\delta}_\mathrm{c}}\ge 1,\end{cases}$$

which is enforced over the entire *x*–*z* plane, where crack propagation is admissible. The yield strength *σ*_Y_ and critical opening *δ*_c_ are related to the fracture toughness *K*_c_ by the following relation:

7 $${K}_\mathrm{c}^{2}\frac{1-{\nu }^{2}}{E}=\frac{{\sigma }_\mathrm{Y}{\delta}_\mathrm{c}}{2}.$$

The cohesive law regularizes the stress singularity at the crack tip over the cohesive zone (Fig. 1b), which has size *X*_c_ and functional shape ${\hat{\tau }}(s)\approx s$ (ref. ^6^):

8 $${X}_\mathrm{c}=\pi \frac{1}{2}\frac{{K}_\mathrm{c}^{2}}{{{\sigma}_\mathrm{Y}}^{2}}{\left(\int_{0}^{1}\frac{\hat{\tau }}{(s)}{\sqrt{s}}\,\mathrm{d}s\right)}^{-2}\approx \pi \frac{9}{32}\frac{{K}_\mathrm{c}^{2}}{{{\sigma}_\mathrm{Y}}^{2}}.$$

We selected *σ*_Y_ and *δ*_c_ so that *X*_c_ ≈ 200 μm, which is lower than all the other relevant length scales.

The initial condition consisted of a straight crack in equilibrium, with applied constant pressure over a region |*x*| < *R*_f_. We modelled the excess critical stress intensity factor at initiation by setting the yield strength beyond the crack front at a lower level than along the front (Supplementary Fig. 4b). Hence, the initial stationary crack of half-length *R* = 10 mm was in an unstable equilibrium. We nucleated a localized rupture by decreasing the yield strength smoothly in space and time over a region of size *r*_nuc_ along the crack front at *x* = *R.* The front at *x* = −*R* was set to remain stationary to avoid any spurious interactions (Supplementary Fig. 4a). Once *r*_nuc_ reached a critical size, the crack propagated towards its stable configuration at *x* = *R* + δ*λ* (Supplementary Video 4 and Supplementary Fig. 4b). The mesh size was such that *X*_c_ was sufficiently discretized. A mesh convergence analysis and sensitivity analysis on boundary conditions were carried out.

## Online content

Any methods, additional references, Nature Portfolio reporting summaries, source data, extended data, supplementary information, acknowledgements, peer review information; details of author contributions and competing interests; and statements of data and code availability are available at 10.1038/s41567-023-02365-0.

### Supplementary information

Supplementary Information Supplementary Figs. 1–5.

Supplementary Video 1 Raw video for *μ* = 400 cP.

Supplementary Video 2 Raw video for all viscosities.

Supplementary Video 3 Derivative video for all viscosities.

Supplementary Video 4 Simulation nucleation and transverse propagation.

## Data Availability

The datasets generated or analysed during the current study are available from the corresponding author upon reasonable request.

## Code availability

The code used to generate the simulation data presented in the manuscript and supplementary materials is available at https://github.com/gabrielealbertini/extended_fracture_uguca_sim.
