# Supplementary material for: Propagation of extended fractures by local nucleation and rapid transverse expansion of crack-front distortion
Source: Nat Phys. 2024 Jan 29;20(4):660–5. doi: 10.1038/s41567-023-02365-0 (PMC11021187; doi:10.1038/s41567-023-02365-0)
Supplement: Supplementary file 1 — Supplementary Figs. 1–5. [file 41567_2023_2365_MOESM1_ESM.pdf]

nature physics

Article

https://doi.org/10.1038/s41567-023-02365-0

Propagation of extended fractures by local nucleation and rapid transverse expansion of crack-front distortion

In the format provided by the authors and unedited

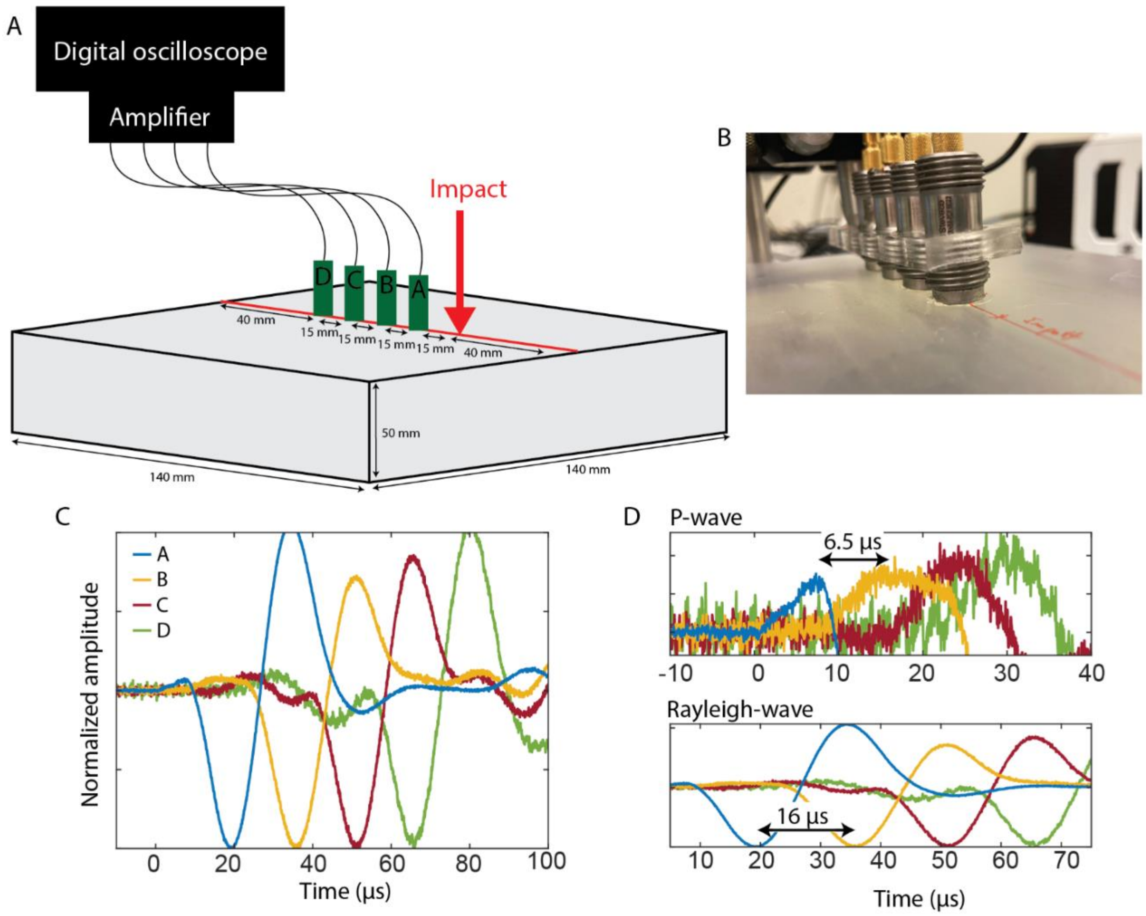

Fig. S1 - Measurement of P-wave, S-wave, and Rayleigh-wave velocities by acoustic methods. A. A metallic ball of 1 mm in diameter is dropped on a 3D printed PMMA sample (140x140x50 mm) from a height of 20 cm, resulting in the generation of acoustic waves. Four acoustic emission sensors (AE) are placed in-line, 15 mm apart from one another. **B.** Photo of the experiment showing the impact point and the four AE sensors. **C.** Raw signal recorded from each of the four AE sensors. **D Top.** From the delay of 6.5 μs between the first-arrival, P-wave signals, we can infer the P-wave velocity, $C_P = 2300\ \mathrm{m/s}$. **D Bottom.** From the delay of 16 μs between the subsequent minima, we can infer the Rayleigh-wave velocity, $C_R = 940\ \mathrm{m/s}$.

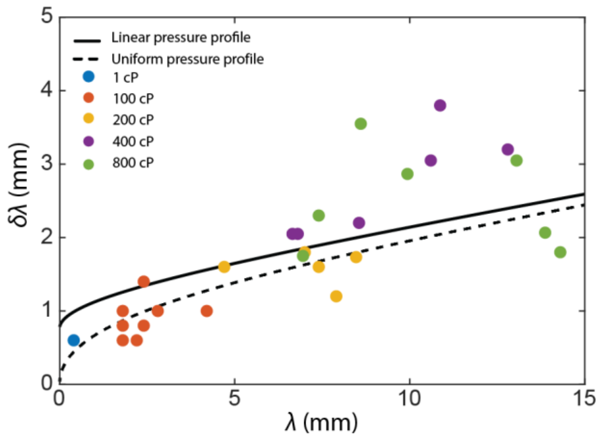

Fig. S2 - Modelling the jump amplitude with different fluid pressure profiles. Measured values (solid points) of $\delta\lambda$ as a function of λ are compared to the predictions of the model (lines). The value of the viscosity of each data point is denoted by a color, given in the legend. To model the break amplitude, we calculate the stress intensity factor (Equation 2), using a linear pressure profile (solid line). For comparison we supplement calculations with a uniform pressure profile (dashed line). The two pressure profiles lead to only slight differences at small values of λ.

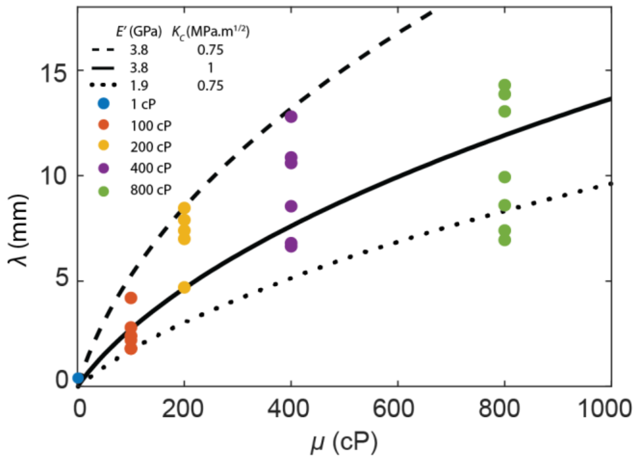

Fig. S3 - Measured dependence of λ on μ compared to the prediction of the model.
Measured values (solid points) of λ as a function of μ compared to the predictions of the model (lines). The value of the viscosity of each data point is denoted by a color, given in the legend. Because the fracture propagates at high velocities, we use $E' = 3.8$ GPa (acoustic measurements). We find a good agreement for a fitted value of $K_c = 1$ MPa $\text{m}^{1/2}$ (solid line). For comparison, we supplement calculation with statically measured values $E' = 1.9$ GPa and $K_c = 0.75$ MPa $\text{m}^{1/2}$ (dotted line). Calculations with a dynamic value of E' and the static measurement of K_c overestimate our results (dashed lines).

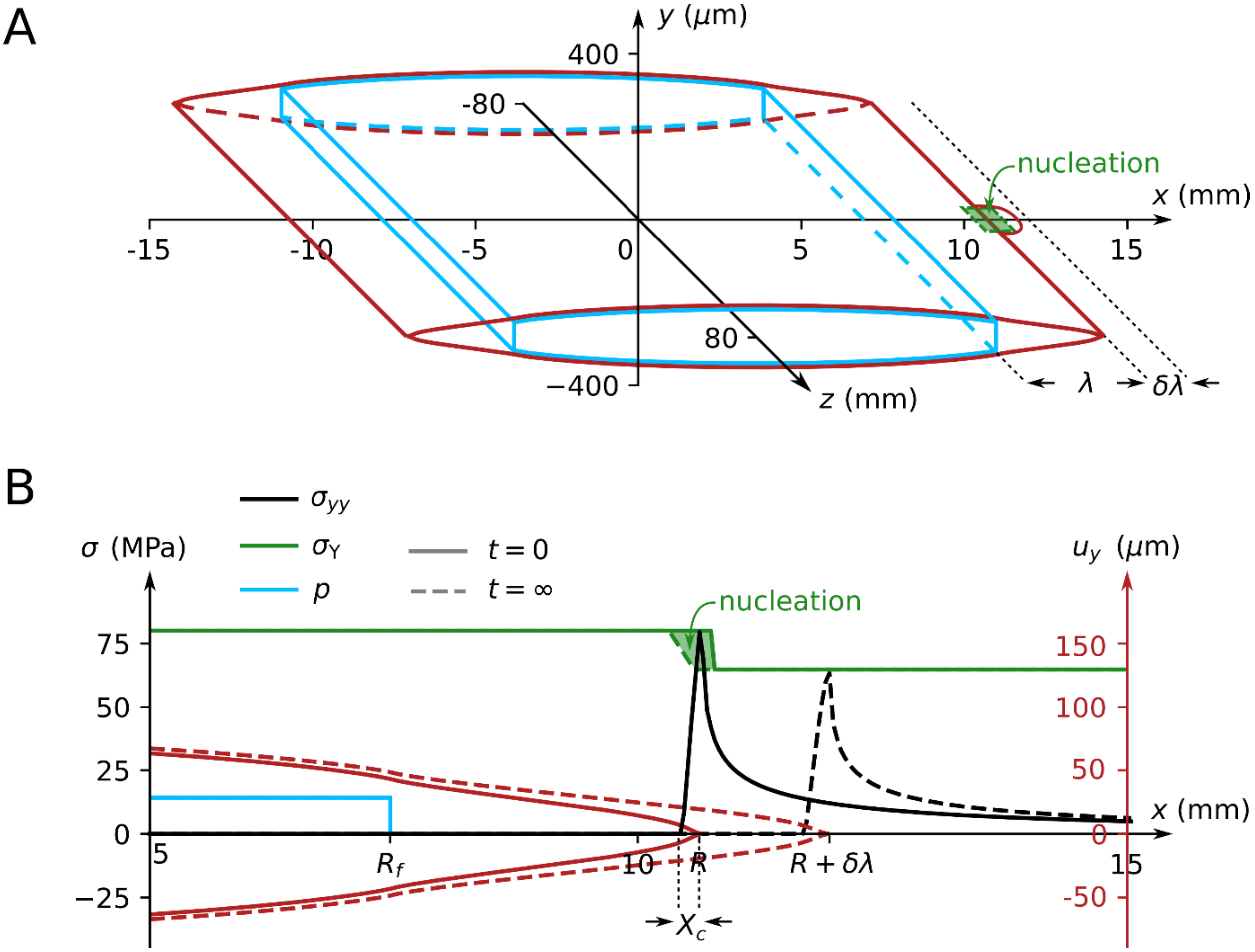

Fig. S4 - Spectral boundary integral simulation set-up.

A. Initial condition and nucleation procedure. The initial crack opening displacement is represented by the red lines. The fluid pressure is uniform and applied in the blue region. The nucleation region is represented by the green shaded area at $x = R$, $y = 0$ and $z = \pm 1$ mm. **B.** Pressure level, p (blue), crack opening displacement, u_y (red), and stress level, σ_{yy} (black), plotted before (solid) and after (dashed) the jump. To nucleate the crack, we locally reduce the yield strength σ_Y in a small region ahead of the crack tip (green shaded area), which results in the crack jumping from R to $R + \delta\lambda$.

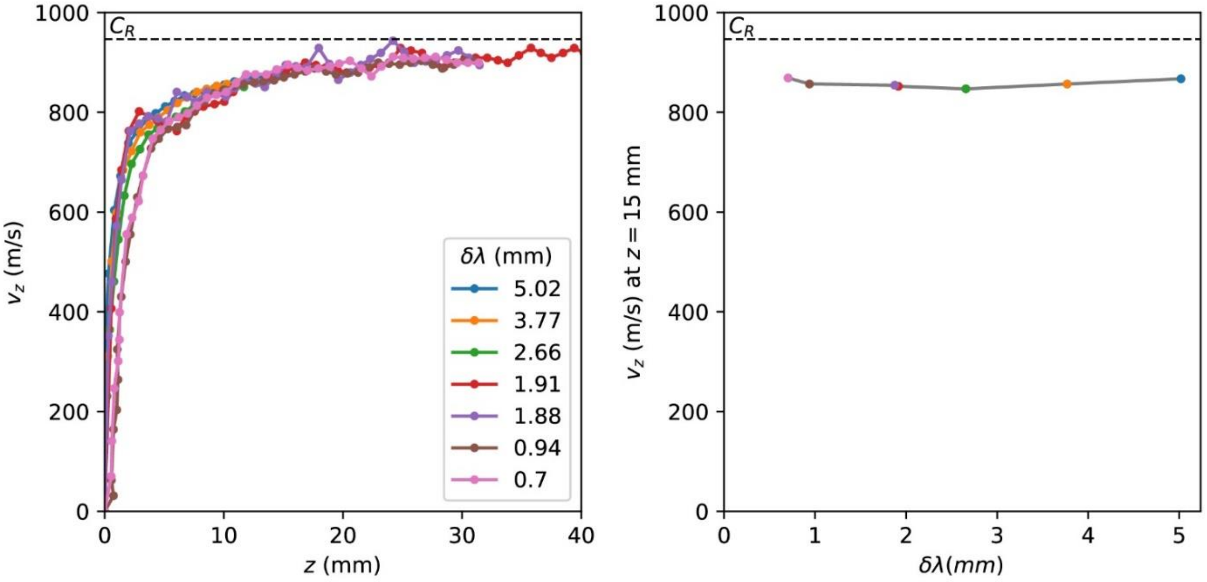

Fig. S5 - Simulated transverse velocity for various jump amplitudes.
A. The simulated transverse velocity, V_z, as a function of the transverse distance, z. **B.** Transverse velocity at a fixed travelled distance, $z = 15$ mm, as a function of the jump amplitude. In the simulation, the transverse velocity profiles are independent of jump amplitude.
